# Transcriptional profiling of the acute pulmonary inflammatory response induced by LPS: role of neutrophils

**DOI:** 10.1186/1465-9921-11-24

**Published:** 2010-02-25

**Authors:** Nejla Güngör, Jeroen LA Pennings, Ad M Knaapen, Roland K Chiu, Marco Peluso, Roger WL Godschalk, Frederik J Van Schooten

**Affiliations:** 1 Department of Health Risk Analysis and Toxicology, NUTRIM School for Nutrition, Toxicology and Metabolism, Maastricht University, PO box 616, 6200 MD, Maastricht, the Netherlands; 2 Laboratory for Health Protection Research, National Institute for Public Health and the Environment (RIVM), Bilthoven, the Netherlands; 3 Cancer Risk Factor Branch, ISPO Cancer Prevention and Research Institute, Via Cosimo il Vecchio 2, 50139 Florence, Italy; 4 Schering-Plough, Department of Toxicology and Drug Disposition, PO box 20, 5340 BH, Oss, the Netherlands

## Abstract

**Background:**

Lung cancer often develops in association with chronic pulmonary inflammatory diseases with an influx of neutrophils. More detailed information on inflammatory pathways and the role of neutrophils herein is a prerequisite for understanding the mechanism of inflammation associated cancer.

**Methods:**

In the present study, we used microarrays in order to obtain a global view of the transcriptional responses of the lung to LPS in mice, which mimics an acute lung inflammation. To investigate the influence of neutrophils in this process, we depleted mice from circulating neutrophils by treatment with anti-PMN antibodies prior to LPS exposure.

**Results:**

A total of 514 genes was greater than 1.5-fold differentially expressed in the LPS induced lung inflammation model. 394 of the 514 were up regulated genes mostly involved in cell cycle and immune/inflammation related processes, such as cytokine/chemokine activity and signalling. Down regulated genes represented nonimmune processes, such as development, metabolism and transport. Notably, the number of genes and pathways that were differentially expressed, was reduced when animals were depleted from circulating neutrophils, confirming the central role of neutrophils in the inflammatory response. Furthermore, there was a significant correlation between the differentially expressed gene list and the promutagenic DNA lesion M_1_dG, suggesting that it is the extent of the immune response which drives genetic instability in the inflamed lung. Several genes that were specifically regulated by the presence of activated neutrophils could be identified and these were mostly involved in interferon signalling, oxidative stress response and cell cycle progression. The latter possibly refers to a higher rate of cell turnover in the inflamed lung with neutrophils, suggesting that the neutrophil influx is associated with a higher risk for the accumulation and fixation of mutations.

**Conclusion:**

Gene expression profiling identified specific genes and pathways that are related to neutrophilic inflammation and could be associated to cancer development and indicate an active role of neutrophils in mediating the LPS induced inflammatory response in the mouse lung.

## Background

Inflammation is often considered to be a critical component of tumourigenesis, since many cancers arise at sites of infection, chronic irritation and inflammation [1,2]. Also subjects suffering from inflammatory pulmonary diseases, such as chronic obstructive pulmonary disease (COPD)/emphysema, have an increased risk for developing lung cancer [3]. One common characteristic of many inflammatory lung disorders is the influx of polymorphonuclear neutrophils (PMN), which are highly specialised members of the innate immune system. Neutrophils are often the first immune cells to arrive at sites of infection and their primary function is to phagocytise and destroy invading microorganisms and/or foreign material. Under normal conditions, neutrophils circulate through the bloodstream, having a half life span of about 7 hours. However, during an invasion of pathogens, neutrophils are recruited to the site of inflammation and their life span is increased up to 4 days [4]. It has been suggested that the accumulation of these neutrophils in the lumen of the lung is related to lung cancer risk [5], implying a significant role of the neutrophilic inflammation in the carcinogenic response [6].

Inflammation is considered to initiate and promote lung cancer development *via *the continuous formation of reactive oxygen or nitrogen species (ROS/RNS) that can bind to DNA, and thus lead to promutagenic DNA alterations [7,8]. Furthermore, the release of ROS by neutrophils is also believed to cause a cell proliferative response, which may contribute significantly to the carcinogenic response following chronic inflammation. We have previously reported two distinct pathways of neutrophil induced genotoxicity and mutagenicity *via *their capacity to release the oxidant hypochlorous acid (HOCl). Exposure of lung epithelial cells to HOCl leads to: (i) indirect DNA disturbances by attack of HOCl on lipids/proteins, resulting in the formation of promutagenic DNA adducts [9] and (ii) impaired removal of bulky DNA lesions due to inhibition of nucleotide excision repair (NER) [10,11]. Although both processes are thought to play an important role in tumourigenesis, additional processes may be involved to create a microenvironment that facilitates tumour formation and progression. Thus, a better understanding of biochemical pathways in the lung elicited by inflammatory stimuli, and the role of neutrophils herein, is required to further unravel mechanisms of inflammation related lung carcinogenesis.

In search for the central role of neutrophils in lung inflammation and associated cancer development, we determined the transcriptional response upon neutrophilic lung inflammation in lung cells of mice treated intratracheally with lipopolysaccharide (LPS), by identifying genes that are differentially expressed. LPS, the well characterised component of gram-negative bacterial cell walls was used, as it is a potent endotoxin capable of inducing a strong inflammatory response by recruiting circulating PMN to the lungs [12]. Indeed, intratracheal instillation of experimental animals with LPS induces an acute lung inflammation associated with a neutrophil influx into the alveoli [13]. We further demonstrated the specific role of neutrophils by antibody depletion of circulating neutrophils from the animals. In this report, we present the profiles of differentially expressed genes in lung tissue due to neutrophilic inflammation and investigated whether neutrophil induced genes are associated with induction of genotoxicity. Moreover, we related the biological networks/pathways including these genes to the potential role of neutrophils in pulmonary inflammation related carcinogenesis.

## Methods

### Animals

Male C57Bl6 mice (~12 weeks old) were obtained from Charles River Breeding Laboratories (Heidelberg, Germany). Mice were housed individually in standard laboratory cages and allowed food and water *ad libitum *throughout the experiments. The studies were carried out in accordance with an approved protocol by the Institutional Animal Care Committee of Maastricht University.

### LPS induced acute lung inflammation mouse model

Mice were exposed to LPS (*Escherichia Coli*, serotype O55:B5, Sigma, St. Louis, MO, USA) by intratracheal instillation to induce an acute pulmonary inflammation. The used dose of LPS was 20 μg/instillation/mouse. Intratracheal instillation was performed by a nonsurgical technique under anaesthesia as previously described [14]. Sham mice (n = 5) were instilled with sterile 0.9% NaCl. Since a previous study [13] demonstrated that local LPS challenge in C57Bl6 mice results in a time dependent neutrophil accumulation peaking at day 3, mice (n = 5) were sacrificed 3 days postexposure by 115 mg/kg sodium pentobarbital (Ceva Sante Animale, Maassluis, the Netherlands) and bronchoalveolar lavage (BAL) (3× with 1 ml sterile 0.9% NaCl) was performed to remove inflammatory cells from the airways. After centrifugation at 1,500 rpm during 10 min at 4°C, the cell-free BAL fluid (BALF) was stored at -80°C for MPO activity measurement. Lavaged lungs were snapfrozen and pulverised using a mortar and pestle. The pulverised lung tissue was stored at -80°C for DNA, RNA and protein isolation.

To unravel the role of neutrophils in the inflammatory airway transcriptome, depletion of circulating neutrophils in mice (n = 5) was achieved by intraperitoneal injection of 0.5 mg of the monoclonal rat anti-mouse neutrophil antibody NIMP-R14 (Hbt, Uden, the Netherlands), 24 h before i.t. LPS instillation. NIMP-R14 selectively depletes mouse neutrophils *in vivo *for up to 6 days [15,16]. Again, 3 days after instillation, mice were sacrificed, and lungs were lavaged and isolated as described above. In our study, a lack of MPO activity in the lung was used as a verification of the absence of neutrophils. IgG (eBioscience, San Diego, CA, USA) was used as control mAb in the sham group and LPS group without neutrophil depletion.

### MPO activity measurement

MPO packaged in neutrophils will have no effect on pulmonary epithelial tissue, because MPO must be released extracellularly during the oxidative burst of neutrophils. Therefore, extracellular MPO activity was measured in cell-free BALF as described by Klebanoff *et al*. [17] and is indicative for the presence of activated neutrophils in the lung [18].

### MPO ELISA

The presence of residual inflammatory neutrophils in the lavaged lung may affect the analysis of the transcriptome of lung epithelial cells. Therefore, to evaluate the degree of contamination of the lavaged lungs with resident neutrophils, proteins were extracted and MPO protein levels were determined quantitatively in lung homogenates, using a mouse MPO ELISA kit (Hbt, Uden, the Netherlands) according to the manufacturer's instructions. Protein concentrations were determined spectrophotometrically, using the DC-protein Assay Kit (BIORAD, Veenendaal, the Netherlands). The amount of MPO in the lung tissue was expressed as ng MPO/mg protein.

### DNA isolation

DNA was isolated using standard phenol extraction [19]. DNA concentrations were quantified by NanoDrop (Isogen-Lifescience, De Meern, the Netherlands) and samples were frozen at -20°C until further analysis.

### Quantification of M_1_dG

The ^32^P-postlabelling technique was used to analyse the levels of 3-(2-Deoxy-*β*-D-*erythro*-pentofuranosyl)pyrimido [1,2-*α*]purin-10(3*H*)-one (M_1_dG) adducts, as previously reported [20]. M_1_dG adduct analysis was carried out by PEI-cellulose TLC chromatography according to published conditions [21]. Detection and quantification of M_1_dG adducts and total nucleotides were obtained by phosphor imaging technology (Typhoon 9210, Amersham) and ImageQuant software (Molecular Dynamics, Sunnyvale, CA, USA). After background subtraction, the levels of DNA adducts were expressed as relative adduct labelling (RAL = adducted nucleotides/total nucleotides). Standard MDA modified [22] and unmodified DNA were routinely processed in the analysis as controls.

### RNA isolation and purification

After lungs were pulverised using a liquid nitrogen cooled mortar and pestle, TRIzol^® ^Reagent (Invitrogen, Breda, the Netherlands) was added and RNA was isolated by using an RNeasy Mini kit (Qiagen, Venlo, the Netherlands) with DNase treatment, according to the manufacturer's protocol. RNA quantity was determined by NanoDrop (Isogen-Lifescience, De Meern, the Netherlands) and RNA quality was assessed by automated gel electrophoresis on an Agilent 2100 Bioanalyser (Agilent Technologies, Amstelveen, the Netherlands). All RNA samples analysed were pure and free of RNA degradation.

### RNA labelling and microarray hybridisation

Cyanine labelled cRNA was generated by using the Two-Colour Microarray based Gene Expression Analysis kit from Agilent Technologies. All samples were labelled with Cy5 and a pool of Cy3 labelled sham group samples was used as a reference. Dye incorporation rates were used to put appropriate amounts of Cy5 and Cy3 labelled samples (10 pmol each) together for hybridisation on Agilent 4× 44 K Mouse Microarrays (Agilent Technologies). After hybridisation, slides were washed and dried with N_2 _gas before scanning.

### Image analysis

Slides were scanned on a GenePix^® ^4000B Microarray Scanner (Molecular Devices, Sunnyvale, CA, USA). Cy5 and Cy3 were excited at wavelengths of 635 and 532 nm respectively. Laser power was set to 100%. The photo multiplier was set to a saturation tolerance of 0.02% to minimise background and saturated spots. The obtained images (resolution 5 micron, 16 bit tiff images) were processed with Imagene 8.0.1 software (Biodiscovery, El Segundo, CA, USA) to measure median Cy3 and Cy5 signal intensities for spots and local backgrounds. Quality control was performed by means of visual inspection of the scanned images, raw data scatter plots, MA plots and normal probability plots to assess signal distribution. Positive and negative controls were used for quality control, but subsequently excluded from the further normalisation and analysis.

### Normalisation and data preparation

Raw microarray signal data were normalised in R http://www.r-project.org, using the four step approach as described by Baken *et al*. [23]: (1) natural log-transformation, (2) quantile normalisation of all scans, (3) correcting the sample spot signal for differences in the corresponding reference spot signal between arrays and (4) averaging data from spots annotated with an identical gene symbol. Normalised data for the resulting 25,696 genes were visualised by Principal Component Analysis (PCA) for additional quality control. Further analyses were carried out in R and Microsoft Excel. As PCA analysis showed heterogeneity within groups, for each gene, median expression values per group were calculated and compared between the 3 experimental groups. Genes with a more than 1.5-fold difference between experimental groups were considered differentially expressed. It has been described by Guo *et al*. [24] that for such strong effects, analyses of such gene lists showed good and internal consistency on the Gene Ontology and pathway level.

### Data analysis

Functional Annotation and Gene Ontology (GO) term enrichment were examined with DAVID Bioinformatics Resource http://david.abcc.ncifcrf.gov/[25]. Additionally, MetaCore (GeneGO, San Diego, CA) was used for additional pathway enrichment analysis, as well as visualisation of differentially expressed genes in cellular pathways.

To provide a sample specific measure for the gene expression response over the differentially expressed genes, their value was adjusted for the median of the control (sham) group, followed by calculation of the root mean square.

Correlation between the gene expression response and M_1_dG DNA adduct lesions were compared between samples using the Spearman's rank correlation coefficient.

## Results

### Neutrophil infiltration in lung tissue

In order to determine the influx of neutrophils and to verify neutrophil activation, we assessed MPO activity in the BALF. Extracellular MPO is indicative of the presence of activated neutrophils [18]. Intratracheal instillation of mice with LPS indeed increased the mean MPO activity in BALF (81.4 ± 20.5 mU/ml), as compared to the sham group (MPO activity < detection limit). MPO activity was also absent in animals depleted of circulating neutrophils, thereby confirming their depleted status.

Furthermore, MPO protein concentrations in lung tissue homogenates, prepared after BAL, were determined to check the efficiency of the BAL and the possible contamination of the lavaged lungs with resident neutrophils, and hence neutrophilic RNA. MPO protein values in the lavaged lung tissues of LPS exposed mice with PMN (145.99 ± 94.82 ng MPO/mg protein, after correction for background levels in LPS exposed neutrophil depleted mice) indicated no significant neutrophilic contamination compared to sham mice (58.18 ± 77.26 ng MPO/mg protein). The mean MPO protein content of lung samples of LPS exposed mice with PMN that were not lavaged (obtained from another study) was 546.5 ± 284.6 ng MPO/mg protein and significantly higher then the MPO protein level in sham mice, which indicates that the lavages were successful, washing away most of the neutrophils/inflammatory cells and the microarray analysis will thus not be disturbed by remaining infiltrated neutrophils cells.

### Differentially expressed genes

PCA analysis on normalised data showed that LPS exposed groups were more heterogeneous than the sham group. This was also the case in a PCA using only the 383 commonly lung inflammation genes that were described, by Pennings *et al*., to be induced in multiple lung inflammatory models [26], indicating that also the inflammatory response, which is the main expected response, is subjected to relatively large inter-animal variation following LPS exposure. This observation is additionally reflected by the heterogeneity in MPO activity levels measured in BALF and is probably a consequence of the differences in the acute phase response of individual mice upon intratracheal LPS instillation. To render the gene expression data analysis robust against this heterogeneity and outliers within groups, we based our further analysis on the median values for each group and only focussed on the genes with the highest fold change ranking only.

**Figure 1:**
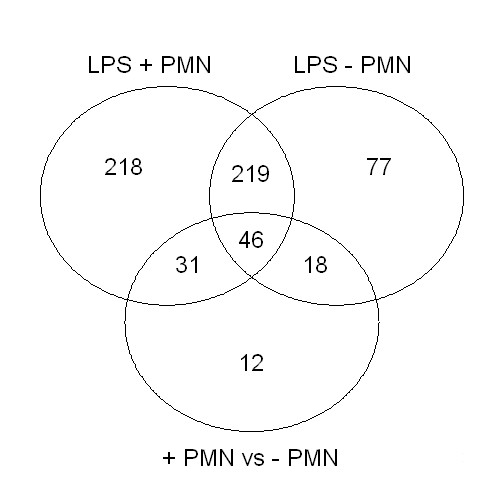
**Venn diagram showing the distribution of the 1.5-fold differentially expressed genes over the three groups**: LPS exposed mice compared to sham mice (LPS + PMN), LPS exposed neutrophil depleted mice compared to sham mice (LPS -PMN) and LPS exposed mice compared to LPS exposed neutrophil depleted mice (+ PMN *vs*. - PMN).

This approach revealed 621 genes that were differentially expressed between all experimental groups with a ≥ 1.5-fold change (See additional file 1: List of genes differentially expressed more then 1.5-fold upon LPS instillation in the lung), of which 179 were > 2-fold differentially expressed. The distribution of the number of differentially regulated genes over the experimental groups is presented in a Venn diagram (Fig. 1). The expression of 514 genes was changed in the LPS group in the presence of PMN, of which 394 showed increased expression and 120 decreased expression as compared to sham control. The greatest increase was 15-fold (serum amyloid A 3) and the greatest decrease was 2.6-fold (paraoxonase 1). In total, 360 genes were differentially expressed in the LPS group that was depleted of PMN (287 upregulated and 73 downregulated). The greatest increase in this list is 7.7-fold, again for serum amyloid A 3, and the greatest decrease was 2.7-fold (keratin complex 1, acidic gene 15). As demonstrated in Figure 1, there were overlaps between the gene lists obtained from LPS exposed animals with and without PMN depletion. Among the differentially expressed genes compared to sham mice, 265 were overlapping both groups, showing differential regulation upon LPS induced lung inflammation, irrespective of the neutrophil influx status. A correlation (*R *= 0.68) was observed between the expression of these genes in animals that were exposed to LPS with or without depletion of PMN. However, the slope of the regression line was 0.6, indicating that the extent of the response was less strong in animals that were PMN depleted. Comparison of the 2 LPS exposed groups with/without PMN depletion resulted in 107 differentially expressed genes, mostly overlapping the lists of differentially expressed genes of the separate LPS groups compared to the sham group, but different in the extent of change. Only 12 genes were differentially expressed by PMN depletion and not by LPS instillation, of which only 1 gene was 2-fold changed (*i.e*. resistin like gamma).

### Pathway regulation

To assign functions to differentially expressed genes, we classified them according to GO categories. Subsequently, enrichment of GO categories in the list of 1.5-fold differentially expressed genes was assessed to identify pathophysiological processes involved in the acute phase response in the lung upon LPS induced lung inflammation and the role of neutrophils herein. As expected, this GO term enrichment analysis showed that, among the 514 genes differentially expressed in the LPS exposed group in the presence of PMN, a considerable number of the 394 upregulated genes belonged to the GO categories "immune response" (74 genes) and "inflammatory response (41 genes). The functional classes of genes that were most prominently upregulated consisted of cytokines and chemokines, several genes involved in the complement cascade and a few known inflammation markers (*e.g. Lcn2, Saa1, Saa3*). Apart from genes involved in immune or inflammation related processes, a substantial number of differentially regulated genes (46 genes) is involved in cell cycle regulation. GO categories belonging to the 120 downregulated genes mostly represented nonimmune processes, such as development, metabolism and transport. Depleting the mice from circulating neutrophils resulted in a comparable but weaker immune/inflammatory response upon LPS exposure. Moreover, the number of genes belonging to the GO categories "immune response" and "inflammatory response" was reduced from 74 to 59 and from 41 to 29 genes, respectively, due to PMN depletion. Also the number of genes belonging to the GO category cell cycle was reduced to 36 genes in PMN depleted mice.

To reveal intergene relations, we performed MetaCore analysis on the 1.5-fold regulated genes upon LPS exposure in the combined group with or without PMN depletion. The significant pathways are presented in Table 1. This analysis revealed a common significance of pathways involved in cell cycle for both LPS exposed groups, although cell cycle effects were more pronounced in the presence of neutrophils. Further, a large number of pathways involved in immune response were modulated upon LPS exposure. High ranking MetaCore pathways in both LPS groups were "Alternative complement pathway" and "Antigen presentation by MHC class II". Interestingly, several immune response pathways, including the classic and lectin induced complement cascade, interferon and IL-10 signalling, were only significantly induced in the LPS exposed group without PMN depletion. Whereas the transcriptional regulation of myeloid progenitor cell differentiation into neutrophils was only modulated in the LPS exposed group with PMN depletion, as indicated by higher *Mpo *expression in this group. The rest of the differentially expressed inflammation associated transcripts upon LPS exposure in depleted and undepleted mice were categorised into the following cellular processes: oxidative stress, apoptosis, G protein signalling, cytoskeleton remodelling and metabolism. As shown in Table 1, the overall response is rather similar in both LPS groups compared to sham group, but generally less extensive in the group in which PMN were depleted, as indicated by fewer modulated pathways and fewer genes involved in these pathways.

**Table 1:** Pathways significantly modulated by LPS induced lung inflammation as found in MetaCore

Main cellular process	Modulated pathways	*P *value		Genes in pathway		
		
				Expressed		total
					
		+ PMN	- PMN	+ PMN	- PMN	
Cell cycle	Role of APC in cell cycle regulation	1,040E-09	8,149E-08	15	12	32
	Chromosome condensation in prometaphase	4,131E-06	8,392E-11	9	12	20
	The metaphase checkpoint	4,423E-06	1,474E-04	12	9	36
	Spindle assembly and chromosome separation	3,170E-04	1,937E-03	9	7	32
	Start of DNA replication in early S phase	1,284E-03	3,115E-02	8	5	31
	Initiation of mitosis	1,544E-03	2,483E-03	7	6	25
	Sister chromatid cohesion	1,530E-02		5		21
	Transition and termination of DNA replication		1,523E-02		5	26
	Role of Nek in cell cycle regulation		2,390E-02		5	29
	Nucleocytoplasmic transport of CDK/Cyclins		4,386E-02		3	14
Immune response	Alternative complement pathway	4,539E-07	2,737E-02	12	5	30
	Fc gamma R-mediated phagocytosis	1,606E-03	9,058E-03	8	6	32
	Antigen presentation by MHC class II	6,046E-03	2,644E-03	4	4	11
	Classic complement pathway	1,517E-05		12		40
	Antiviral actions of Interferons	2,431E-04		9		31
	CCR3 signalling	8,728E-04		12		59
	Lectin Induced complement pathway	1,251E-03		9		38
	Lipoxin inhibitory action on Superoxide production	1,544E-03	2,483E-03	7	6	25
	IFN alpha/beta signalling pathway	6,214E-03		6		24
	IL-10 signalling pathway	2,245E-02		5		23
	Antigen presentation by MHC class I	3,675E-02		5		26
	Transcription regulation of granulocyte development		3,115E-02		5	31
Oxidative stress	ROS production	8,932E-04	4,113E-02	7	4	23
Apoptosis	Inhibition of ROS induced apoptosis	3,675E-02		5		26
G protein signalling	Rac2 regulation pathway	4,957E-03	4,113E-02	6	4	23
	RAC1 in cellular process	1,361E-02		6		28
Cytoskeleton remodelling	Regulation of actin cytoskeleton by Rho GTPases	8,972E-03			5	23
	Alpha-1A adrenergic receptor-dependent inhibition of PI3K	2,887E-02			3	12
Metabolic process	Lipoprotein metabolism I. Chylomicron, VLDL and LDL metabolism	1,630E-02	9,007E-07	3	6	8
	Lipoprotein metabolism II. HDL metabolism	1,630E-02	9,007E-07	3	6	8
	G-alpha(q) regulation of lipid metabolism	2,245E-02		5		23
	Urea cycle	3,675E-02		5		26
	LDL metabolism during development of fatty streak lesion	1,870E-02			2	4

Note: Significantly modulated 1.5-fold changed genes (P value < 0.05) versus total number of genes in pathway

Biological processes and pathways for differentially regulated genes between the PMN depleted and undepleted group (both with LPS exposure) were also identified by querying the MetaCore database. The significant pathways, listed in Table 2, primarily reveal a role of inflammatory neutrophil influx in immune response processes, such as interferon, IL-3 and Oncostatin M signalling.

**Table 2:** Pathways significantly modulated by neutrophil depletion during LPS induced lung inflammation as found in MetaCore

Main cellular process	Modulated pathways	*P *value	Genes in pathway	
			
			expressed	total
Cell cycle	Role of APC in cell cycle regulation	1,012E-02	4	32
	Spindle assembly and chromosome separation	1,012E-02	4	32
Immune response	IFN alpha/beta signalling pathway	2,740E-05	6	24
	Alternative complement pathway	8,024E-03	4	30
	Oncostatin M signaling via MAPK	1,683E-02	4	35
	NKG2D signalling	4,297E-02	3	29
	IL-3 activation and signallng pathway	4,685E-02	3	30
Response to extracellular stimulus	Angiotensin signalling via STATs	2,917E-02	3	25
	Ligand dependent activation of the ESR-1/AP-1 pathway	4,756E-02	2	13
Other	Mucin expression in CF via IL-6, IL-17 signalling pathways	3,926E-02	3	28

Note: Significantly modulated 1.5-fold changed genes (P value < 0.05) versus total number of genes in pathway

### Correlation of genes with phenotypic markers of effects; M_1_dG adducts

Considering the fact that the pathway analysis tool MetaCore only identified a significant modulation of oxidative stress related pathways upon LPS instillation in the group without PMN depletion (see Table 1), we sought to link neutrophil induced gene expression changes with phenotypic genotoxic effects of neutrophil derived ROS. As a previous study indicated that neutrophils are possibly mutagenic *via *an indirect genotoxic hazard of neutrophil derived HOCl, by its attack on lipids and/or proteins producing MDA and forming M_1_dG adducts [9], we measured M_1_dG levels in the mouse lung as a phenotypic marker of neutrophil induced genotoxicity. LPS treatment caused a significant increase in the formation of M_1_dG adducts in the mouse lung (13.6 ± 2.8 M_1_dG/10^8 ^nt) compared to sham mice (5.1 ± 0.2 M_1_dG/10^8 ^nt) (~4-fold, *P *= 0.01). Reduced M_1_dG levels in LPS instilled neutrophil depleted mice (10.6 ± 2.5 M_1_dG/10^8 ^nt), and a significant correlation between MPO activity in BALF and M_1_dG levels in lung cells (Pearson correlation = 0.67, *P *= 0.001) suggest a direct link between neutrophil influx, MPO mediated formation of HOCl and formation of M_1_dG adducts *in vivo*. Therefore, we calculated Spearman's rank correlation coefficient between gene expression response values and M_1_dG adduct data, indicating a significant correlation with *R *= 0.64 and *P *= 0.01, using the 1.5-fold changed gene lists.

## Discussion

Gene expression changes usually occur early during disease development and can provide additional information on the pathogenesis of that particular disease. The present study makes use of the ability of microarray technology to define the expression of inflammation related genes in lungs, by using LPS in a mouse model combined with systemic neutrophil depletion. In this way, we were able to extend the understanding of the role of neutrophils in pulmonary inflammation and identify genes and pathways that can be related to mechanisms of inflammation related carcinogenesis.

As LPS induced pulmonary inflammation involves a massive recruitment of neutrophils into the alveoli, it raises the question whether the observed transcriptional responses in sessile lung cells can actually be attributed to the fact that the lung samples are contaminated with resident inflammatory neutrophils. However, inflammatory cells were removed from the lungs prior to expression analysis, and similar MPO protein levels in sham and LPS exposed animals indicated efficient BAL and neglectable levels of resident neutrophils. Thus, the changes in gene expression described here can predominantly be attributed to sessile pulmonary epithelial cells.

Among the 1.5-fold differentially expressed genes, the number of differentially expressed genes in the group of LPS exposure without neutrophil depletion was larger then those in the LPS group with neutrophil depletion, indicating a more pronounced response when LPS induced lung inflammation is associated with neutrophil influx in terms of changes of gene expression (Fig. 1). The reduced acute inflammatory response in the neutrophil depleted animals was also confirmed by lower expression levels of the serum amyloid genes *Saa1 *and *Saa3*, which are acute phase systemic inflammation markers known to be induced by LPS treatment [27]. Overall, there is a consistency of changed gene expression profiles between LPS exposed animals with or without neutrophil depletion, which is reflected by the fact that 74% of the > 1.5-fold changed genes in the LPS group with neutrophil depletion were overlapping the LPS group without neutrophil depletion. In comparison, a high percentage of differentially expressed genes between PMN depleted and undepleted group (89%) were also found in the gene sets of which the expression changed in the exposed groups as compared to sham, indicating that neutrophil specific gene profile changes in the lung were almost only noticeable after LPS exposure.

The shared response of undepleted mice and neutrophil depleted mice upon LPS exposure involves predominantly increased expression of cell cycle related genes. Apparently there is a rapid turnover or proliferation of cells due to increased lung epithelial renewal, which is actually often seen during acute lung inflammation [26]. As expected, besides this modulation of cell cycle, most upregulated genes are involved in immune and inflammation related processes, such as cytokine/chemokine activity and signalling, complement cascade and antigen presentation. Other modulated processes include oxidative stress response, apoptosis, cytoskeleton remodelling and lipid metabolism. Most of the genes that were down regulated by LPS exposure involved nonimmune processes, such as development, metabolism and transport. It is tempting to speculate that down regulation of these processes shifts the energy balance in favour of the immune response, which suggests that the induction of the inflammatory response goes at the expense of normally activated processes in lung tissue. Comparison of our data to several reported lung inflammatory models indicated a comparable trend for the regulation of the set of commonly lung inflammation regulated genes, described by Pennings *et al*. [26].

Evidently, influx of inflammatory cells in the lung is likely to influence gene expression profiles. Pathway analysis revealed restrained cell cycle progression in the neutrophil depleted animals by modifications in cell cycle regulation, DNA replication and metaphase checkpoints, possibly referring to a higher rate of cell turnover in the undepleted LPS exposed group, suggesting that the neutrophil influx is associated with a higher risk for the accumulation and fixation of mutations. In addition, our data provide more information on the central role of PMN in the lung inflammatory response. Pathogenesis of LPS induced acute lung inflammation is characterised by a profound neutrophil influx [12], as also confirmed by pathway analysis in this study, showing the most significant induction of relevant pathways in the LPS induced inflammatory response in the group without PMN depletion. The reduced amount of genes involved in inflammatory/immune processes in the LPS group with PMN depletion can be related to the absence of neutrophils and consequently restrained lung inflammation. This suppressive effect of neutrophil depletion is the most obvious in the expression of multiple genes involved in pathways related to interferon signalling, such as the "IFNα/β signalling pathway" and the "NK62D signalling pathway". By depleting neutrophils, the interferon signalling pathways are down regulated, indicating a reduced level of lung inflammation because interferon signalling is known to play a role in the regulation of the inflammatory response in the lower respiratory tract after LPS inhalation [28]. The expression of genes involved in the "NK62D signalling pathway", resulting in GM-CSF and IFN-γ production, is, however, significantly upregulated in neutrophil depleted mice. GM-CSF is a proinflammatory cytokine, also synthesised by lung epithelial cells, which promotes the growth and maturation of granulocyte progenitors and the activation of mature neutrophils [29]. It primes neutrophils, making them more receptive to activation by secondary stimuli [30]. Knowing this role of GM-CSF, and the fact that several cytokines and chemokines are regulated by IFN-γ, it is conceivable that in the absence of normal PMN recruitment, homeostatic mechanism may serve to promote differentiation of myeloid progenitor cells into mature neutrophils and to enhance the production and release of cytokines after inhalation of LPS. Among the differentially regulated genes in these pathways are *Stat2 *and some interferon induced genes, such as *Ifit2 *and *Isg15*, but also genes involved in AP-1 signalling, for instance the 1.5-fold upregulation of *Fos *expression upon neutrophil depletion of LPS treated mice. c-Fos levels regulate the transcriptional activity of the transcription factor activator protein 1 (AP-1), which is a heterodimer of the proto-oncogene proteins c-FOS and c-JUN [31]. Interestingly, the promoter regions of many inflammatory cytokines contain AP-1 binding sites [32], suggesting that AP-1 activation may be necessary for the induction of acute cytokine mediated inflammation. In this way, c-Fos plays a role in more than one signalling process, interconnecting various signalling routes and thereby extending the inflammatory response. For instance, the interconnection of FOS, AP-1 and STAT in the Oncostatin M signalling pathway, regulates the expression of several acute phase proteins in response to oncostatin M secretion by neutrophils to reduce the local inflammation [33]. Also IL-3 activation involves AP-1; linking the previous mentioned upregulated NK62D signalling pathway to an increased production of IL-3 in neutrophil depleted mice upon LPS induced lung inflammation. IL-3 stimulates the differentiation of multipotent haematopoietic stem cells into myeloid progenitor cells, as well as the proliferation of all cells in the myeloid lineage, including granulocytes, as is confirmed by the observed upregulation of the *Mpo *gene, which is only expressed in neutrophil precursor cells and not in mature neutrophils [34], indicating increased amounts of progenitor cells in the lungs of neutrophil depleted mice. Additionally, the chemoattractants *S100a8 *and *S100a9*, whose expression was induced in LPS exposed neutrophil depleted mice and are known to attract haematopoietic mac1^+ ^myeloid progenitor cells in the lungs [35], may explain this observed accumulation of mac1^+^/gr1^+ ^granulocyte precursors in the lungs of neutrophil depleted mice. Altogether, these modulated pathways in the PMN depleted mice upon LPS exposure can act cooperatively to stimulate the PMN recruitment, suggesting that the organism tries to compensate for the absence of neutrophils.

Furthermore, our study provides evidence that neutrophil influx is involved in transcriptional regulation of oxidative stress during lung inflammation. A network consisting of *Gp91-phox*, *P67-phox*, *P47-phox*, *P22-phox *and *Cytochrome b-558*, was more expressed in LPS group with PMN. Gp91-phox and p22-phox are 2 subunits forming the core heterodimer of NAD(P)H oxidase. NAD(P)H oxidase is one of the major oxidant generating enzymes present in the lung and is induced during an inflammatory status. Imbalance of oxidants/antioxidants has long been hypothesised to play an important role in the pathogenesis of chronic lung inflammation (including COPD) [36] and is associated with cancer development [37]. Moreover, inspection of individual genes in the differentially expressed gene lists showed the upregulation of *Noxo1 *in the LPS group without PMN depletion. NOXO1 is an organiser protein that activates NADPH oxidase (NOX1) [38,39] and increased *Noxo1 *expression may be a marker for oxidative stress during lung inflammation. Together with the neutrophil associated pathway "Inhibition of ROS induced apoptosis", this indicates the important role for neutrophil derived ROS in the induction of DNA damage during acute lung inflammation, which plays a significant part in the carcinogenic process. This was further confirmed by a positive correlation between gene expression changes and the DNA lesion M_1_dG induced by neutrophils during lung inflammation, suggesting that the extent of the inflammatory response is representative for genotoxicity. M_1_dG adducts are efficient promutagenic lesions that induce mutations in genes involved in carcinogenesis [40] and evidence has accumulated that such M_1_dG-DNA adducts play a role in several cancers, notably those with an inflammatory component in their ethiopathogenesis [41]. Thus, this correlation indicates a possible link between neutrophil induced genotoxicity, gene expression changes and initiation of inflammation related cancer. Furthermore, the observed transcriptional response upon an acute lung inflammation may also be addressed to a chronic inflammation, as a chronically higher cell turnover rate associated with genotoxicity suggests that a chronic neutrophil influx is associated with a higher risk of accumulation and fixation of mutations. Moreover, these observations may also add to the known fact that neutrophil infiltration into tumours stimulates tumour progression.

## Conclusions

We used the microarray technology to demonstrate the upregulation of inflammation associated genes in an acute lung inflammation mouse model induced by LPS. Most neutrophil dependent gene expression changes were involved in immune and inflammation related processes. A substantial amount of modulated genes and pathways provided more information on the central role of neutrophils in the inflammatory response to LPS and can impact a variety of seemingly unrelated pathological conditions. For example, sequence changes in innate immune receptors and their signalling molecules are known to alter the risk of developing not only inflammation, but also atherosclerosis and asthma [42]. Thus, the neutrophil associated profiles of the inflammatory response merit further attention, as some of these genes may increase susceptibility to inflammatory lung disease and perhaps cancer [43]. Furthermore, the positive correlation of neutrophil induced genes and the promutagenic DNA lesion M_1_dG, combined with the neutrophil induced upregulation of genes in oxidative stress and cell cycle progression related pathways, suggest that the extent of the inflammatory response drives genetic instability, indicating an active role of neutrophils in tumour initiation and progression.

## Competing interests

The authors declare that they have no competing interests.

## Authors' contributions

NG designed the study and carried out the DNA and RNA isolation, micoarray hybridisation, image analysis and data analysis, and drafted the manuscript. JP carried out the normalisation, data preparation and correlation analysis. AK designed the study and revised the manuscript. RC revised the manuscript. MP carried out the M_1_dG labelling. RG conceived the design of the study, participated in the interpretation of the data and revised the manuscript. FS helped to design the study and revised the manuscript. All authors read and approved the final manuscript.

## Supplementary Material

Additional file 1 List of genes differentially expressed more then 1.5-fold upon LPS instillation in the lung. Click here for file
