# Supplementary material for: Transcriptional profiling of the acute pulmonary inflammatory response induced by LPS: role of neutrophils
Source: Respir Res. 2010 Feb 25;11(1):24. doi: 10.1186/1465-9921-11-24 (PMC2838834; doi:10.1186/1465-9921-11-24)
Supplement: Additional file 1 — List of genes differentially expressed more then 1.5-fold upon LPS instillation in the lung. [file 1465-9921-11-24-S1.PDF]

Appendix to chapter 6: List of genes differentially expressed more then 1.5-fold upon LPS instillation in the lung

symbool	description	Fold change	
		+PMN	-PMN
0610012D17RIK	RIKEN CDNA 0610012D17 GENE	1,54	1,62
1100001G20RIK	RIKEN CDNA 1100001G20 GENE	3,23	3,38
1110003O08RIK	RIKEN CDNA 1110003O08 GENE	-1,61	-1,29
1110069O07RIK	RIKEN CDNA 1110069O07 GENE	-1,68	-1,39
1190002F15RIK	RIKEN CDNA 1190002F15 GENE	1,88	1,92
1190002H23RIK	RIKEN CDNA 1190002H23 GENE	-1,55	-1,17
1190002J23RIK	KLHDC9, KELCH DOMAIN CONTAINING 9	1,56	1,44
1300014I06RIK	RIKEN CDNA 1300014I06 GENE	-1,59	-1,28
1700010A17RIK	RIKEN CDNA 1700010A17 GENE	1,41	1,52
1810010H24RIK	RIKEN CDNA 1810010H24 GENE	-1,69	-1,31
1810011O10RIK	RIKEN CDNA 1810011O10 GENE	-1,54	-1,57
1810033B17RIK	RIKEN CDNA 1810033B17 GENE	2,22	1,99
2010317E24RIK	RIKEN CDNA 2010317E24 GENE	1,56	1,36
2310016F22RIK	RIKEN CDNA 2310016F22 GENE	2,24	1,24
2310043N10RIK	RIKEN CDNA 2310043N10 GENE	-1,60	-1,49
2310067E19RIK	RIKEN CDNA 2310067E19 GENE	-1,65	-1,54
2610002D18RIK	RIKEN CDNA 2610002D18 GENE	1,68	1,60
2610024B07RIK	RIKEN CDNA 2610024B07 GENE	-1,53	-1,46
2810417H13RIK	RIKEN CDNA 2810417H13 GENE	2,74	1,95
2900006A17RIK	RIKEN CDNA 2900006A17 GENE	-1,55	-1,17
2900009J20RIK	RIKEN CDNA 2900009J20 GENE	-1,56	-1,27
3526401B18RIK	AKAP5, A KINASE (PRKA) ANCHOR PROTEIN 5	-1,62	-1,55
4833427B12RIK	GINS2, GINS COMPLEX SUBUNIT 2 (PSF2 HOMOLOG)	2,28	1,52
4930438A08RIK	RIKEN CDNA 4930438A08 GENE	1,04	1,58
5430435G22RIK	RIKEN CDNA 5430435G22 GENE	1,63	1,64
5730416F02RIK	RIKEN CDNA 5730416F02 GENE	1,94	1,69
5730536A07RIK	RIKEN CDNA 5730536A07 GENE	1,70	1,30
6720460F02RIK	RIKEN CDNA 2610008F03 GENE	1,75	1,60
9030425E11RIK	RIKEN CDNA 9030425E11 GENE	1,58	1,08
9130017C17RIK	RIKEN CDNA 9130017C17 GENE	1,68	1,45
9530083O12RIK	RIKEN CDNA 9530083O12 GENE	-1,64	-1,44
A530026G17	HYPOTHETICAL PROTEIN A530026G17	-2,19	-1,56
A630077B13RIK	RIKEN CDNA A630077B13 GENE	1,62	1,37
ACOXL	ACYL-COENZYME A OXIDASE-LIKE	-1,52	-1,57
ACP5	ACID PHOSPHATASE 5, TARTRATE RESISTANT	2,43	1,72
ACTC1	ACTIN, ALPHA, CARDIAC	-1,38	-1,91
ADIPOQ	ADIPONECTIN, C1Q AND COLLAGEN DOMAIN CONTAINING	-1,13	-1,59
AHR	ARYL-HYDROCARBON RECEPTOR	-1,25	-1,59
AI447904	PYHIN1, PYRIN AND HIN DOMAIN FAMILY, MEMBER 1	1,82	1,48
AI451617	EXPRESSED SEQUENCE AI451617	2,06	1,32
AI607873	EXPRESSED SEQUENCE AI607873	2,87	1,87
AIF1	ALLOGRAFT INFLAMMATORY FACTOR 1	2,78	2,21
AK005018	(NO GENE)	1,69	1,45
AK013903	(NO GENE)	1,36	1,56
AK033087	(NO GENE)	-1,54	-1,62
AK035003	(NO GENE)	-1,56	-1,45
AK043317	(NO GENE)	-1,33	-1,57
AK045692	GM4653, PREDICTED GENE 4653	-1,81	-1,27
AK045826	GM14964, PREDICTED GENE 14964	-1,53	-1,12
AK048657	(NO GENE)	-1,50	-1,18
AK081747	(NO GENE)	-1,37	-1,54
AK082620	(NO GENE)	-1,65	-1,36
AK083553	PRODUCT:UNCLASSIFIABLE	-2,11	-1,61
AK083897	(NO GENE)	-1,68	-1,49
AK162987	(NO GENE)	-1,65	-1,51
AKAP12	A KINASE (PRKA) ANCHOR PROTEIN (GRAVIN) 12	-1,63	-1,67
ALDH1A1	ALDEHYDE DEHYDROGENASE FAMILY 1, SUBFAMILY A1	-1,84	-1,38
ALDH6A1	ALDEHYDE DEHYDROGENASE FAMILY 6, SUBFAMILY A1	-1,54	-1,19
ALOX5AP	ARACHIDONATE 5-LIPOXYGENASE ACTIVATING PROTEIN	1,62	1,51
ANKRD47	ANKYRIN REPEAT DOMAIN 47	-1,54	-1,48
ANP32A	ACIDIC (LEUCINE-RICH) NUCLEAR PHOSPHOPROTEIN 32 FAMILY, MEMBER A	-1,52	-1,23
AOX1	ALDEHYDE OXIDASE 1	-1,60	-1,46
APOBEC1	APOLIPOPROTEIN B EDITING COMPLEX 1	1,48	1,61
APOC2	APOLIPOPROTEIN C-II	2,32	1,76
APOD	APOLIPOPROTEIN D	1,94	1,29
APOE	APOLIPOPROTEIN E	1,38	1,55
ARFL4	ARL4D, ADP-RIBOSYLATION FACTOR-LIKE 4D	-1,64	-1,29
ARG1	ARGINASE 1, LIVER	2,81	2,12
ARG2	ARGINASE TYPE II	1,57	1,27
ARL11	ADP-RIBOSYLATION FACTOR-LIKE 11	1,62	1,94
ASF1B	ASF1 ANTI-SILENCING FUNCTION 1 HOMOLOG B (S. CEREVISIAE)	2,63	1,95
ASGR1	ASIALOGLYCOPROTEIN RECEPTOR 1	-1,72	-1,31
ASS1	ARGININOSUCCINATE SYNTHETASE 1	1,66	1,46
ATF3	ACTIVATING TRANSCRIPTION FACTOR 3	1,50	1,58
ATP2B2	ATPASE, CA++ TRANSPORTING, PLASMA MEMBRANE 2	-1,57	-1,54
AURKA	AURORA KINASE A	3,05	2,00
AYTL1	LPCAT2, LYSOPHOSPHATIDYLCHOLINE ACYLTRANSFERASE 2	1,63	1,40
B2M	BETA-2 MICROGLOBULIN	2,11	1,97
BASP1	BRAIN ABUNDANT, MEMBRANE ATTACHED SIGNAL PROTEIN 1	1,40	-1,08
BC004044	CDNA SEQUENCE BC004044	1,59	1,52
BC013712	CDNA SEQUENCE BC013712	1,67	1,20
BC055324	CDNA SEQUENCE BC055324	1,69	1,73

BC071254	(NO GENE)	1,08	1,51
BC089618	PREDICTED GENE 11428	2,32	1,82
BCL2A1B	B-CELL LEUKEMIA/LYMPHOMA 2 RELATED PROTEIN A1B	2,74	1,78
BCL2A1C	B-CELL LEUKEMIA/LYMPHOMA 2 RELATED PROTEIN A1C	2,34	1,82
BEX2	BRAIN EXPRESSED X-LINKED 2	-1,72	-1,27
BIRC1B	NAIP2, NLR FAMILY, APOPTOSIS INHIBITORY PROTEIN 2	2,19	1,60
BIRC5	BACULOVIRAL IAP REPEAT-CONTAINING 5	4,94	3,67
BMPR2	BONE MORPHOGENIC PROTEIN RECEPTOR, TYPE II (SERINE/THREONINE KINASE)	-1,53	-1,49
BRIP1	BRCA1 INTERACTING PROTEIN C-TERMINAL HELICASE 1	1,41	1,50
BST1	BONE MARROW STROMAL CELL ANTIGEN 1	1,99	2,16
BST2	BONE MARROW STROMAL CELL ANTIGEN 2	2,66	1,50
BU531328	(NO GENE)	-1,64	-1,28
BUB1	BUDDING UNINHIBITED BY BENZIMIDAZOLES 1 HOMOLOG (S. CEREVISIAE)	1,61	1,11
BUB1B	BUDDING UNINHIBITED BY BENZIMIDAZOLES 1 HOMOLOG, BETA (S. CEREVISIAE)	1,57	1,72
C130079B09RIK	RIKEN CDNA C130079B09 GENE	-1,47	-1,53
C1QA	COMPLEMENT COMPONENT 1, Q SUBCOMPONENT, ALPHA POLYPEPTIDE	3,31	2,35
C1QB	COMPLEMENT COMPONENT 1, Q SUBCOMPONENT, BETA POLYPEPTIDE	3,39	2,51
C1QC	COMPLEMENT COMPONENT 1, Q SUBCOMPONENT, C CHAIN	2,58	2,27
C1R	COMPLEMENT COMPONENT 1, R SUBCOMPONENT	1,59	1,25
C3	COMPLEMENT COMPONENT 3	1,55	1,35
C4B	COMPLEMENT COMPONENT 4B (CHILDO BLOOD GROUP)	1,65	1,26
CAMP	CATHELICIDIN ANTIMICROBIAL PEPTIDE	-1,02	2,95
CAPG	CAPPING PROTEIN (ACTIN FILAMENT), GELSOLIN-LIKE	2,16	1,70
CAR13	CARBONIC ANHYDRASE 13	1,99	1,72
CAR3	CARBONIC ANHYDRASE 3	-1,02	-1,56
CASC5	RIKEN CDNA 5730505K17 GENE	1,54	1,30
CASP4	CASPASE 4, APOPTOSIS-RELATED CYSTEINE PEPTIDASE	1,53	1,31
CBX7	CHROMOBOX HOMOLOG 7	-1,65	-1,20
CCDC67	COILED-COIL DOMAIN CONTAINING 67	1,70	1,79
CCDC86	RIKEN CDNA 4933411H20 GENE	1,60	1,19
CCDC88	CCDC88B, COILED-COIL DOMAIN CONTAINING 88B	1,54	1,46
CCL2	CHEMOKINE (C-C MOTIF) LIGAND 2	3,61	1,65
CCL3	CHEMOKINE (C-C MOTIF) LIGAND 3	2,03	1,52
CCL4	CHEMOKINE (C-C MOTIF) LIGAND 4	2,15	1,54
CCL6	CHEMOKINE (C-C MOTIF) LIGAND 6	2,32	1,62
CCL7	CHEMOKINE (C-C MOTIF) LIGAND 7	1,70	-1,10
CCL8	CHEMOKINE (C-C MOTIF) LIGAND 8	2,67	2,54
CCL9	CHEMOKINE (C-C MOTIF) LIGAND 9	2,64	1,79
CCNA1	CYCLIN A1	1,55	2,19
CCNA2	CYCLIN A2	3,52	2,43
CCNB1	CYCLIN B1	1,40	1,52
CCNB2	CYCLIN B2	3,69	2,47
CCR5	CHEMOKINE (C-C MOTIF) RECEPTOR 5	2,78	1,93
CD14	CD14 ANTIGEN	1,89	1,59
CD177	CD177 ANTIGEN	1,51	1,66
CD200R1	CD200 RECEPTOR 1	1,40	1,69
CD300D	CD300D ANTIGEN	2,11	1,81
CD300LF	CD300 ANTIGEN LIKE FAMILY MEMBER F	1,87	1,75
CD302	CD302 ANTIGEN	1,56	1,22
CD53	CD53 ANTIGEN	1,64	1,20
CD5L	CD5 ANTIGEN-LIKE	2,07	3,35
CD68	CD68 ANTIGEN	3,02	1,96
CD72	CD72 ANTIGEN	2,65	1,52
CD84	CD84 ANTIGEN	2,31	1,90
CDC20	RIKEN CDNA 2310042N09 GENE	4,03	1,87
CDC2A	CELL DIVISION CYCLE 2 HOMOLOG A (S. POMBE)	1,56	1,76
CDC45L	CELL DIVISION CYCLE 45 HOMOLOG (S. CEREVISIAE)-LIKE	1,54	1,63
CDCA3	CELL DIVISION CYCLE ASSOCIATED 3	2,44	2,03
CDCA5	CELL DIVISION CYCLE ASSOCIATED 5	1,86	1,50
CDCA8	CELL DIVISION CYCLE ASSOCIATED 8	1,83	1,57
CDH16	CADHERIN 16	1,24	-1,32
CDKN3	CYCLIN-DEPENDENT KINASE INHIBITOR 3	2,09	1,90
CDR2L	CEREBELLAR DEGENERATION-RELATED PROTEIN 2-LIKE	-1,19	-1,58
CDT1	RETROVIRAL INTEGRATION SITE 2	1,70	1,29
CENPE	CENTROMERE PROTEIN E	1,92	1,36
CENPF	RIKEN CDNA 6530404A22 GENE	1,62	1,61
CFB	COMPLEMENT FACTOR B	4,97	2,77
CFD	COMPLEMENT FACTOR D (ADIPSIN)	-1,19	-2,26
CFP	PROPERDIN FACTOR, COMPLEMENT	1,53	1,61
CH25H	CHOLESTEROL 25-HYDROXYLASE	2,98	3,16
CHAF1A	CHROMATIN ASSEMBLY FACTOR 1, SUBUNIT A (P150)	2,04	1,51
CHAF1B	RIKEN CDNA 2600017H24 GENE	1,78	1,41
CHI3L1	CHITINASE 3-LIKE 1	1,74	1,43
CHI3L3	CHITINASE 3-LIKE 3	1,78	1,49
CHI3L4	CHITINASE 3-LIKE 4	1,71	1,80
CHIT1	CHITINASE 1 (CHITOTRIOSIDASE)	2,88	2,05
CHMP4B	CHROMATIN MODIFYING PROTEIN 4B	1,51	1,51
CHTF18	CTF18, CHROMOSOME TRANSMISSION FIDELITY FACTOR 18 HOMOLOG (S. CEREVISIAE)	1,67	1,43
CKAP2L	RIKEN CDNA 2610318C08 GENE	1,37	1,94
CKMT2	CREATINE KINASE, MITOCHONDRIAL 2	-1,67	-1,62
CKS1B	CDC28 PROTEIN KINASE 1B	2,35	1,72
CKS2	CDC28 PROTEIN KINASE REGULATORY SUBUNIT 2	2,27	1,63
CLCA3	CHLORIDE CHANNEL CALCIUM ACTIVATED 3	-1,39	-1,76
CLEC3B	C-TYPE LECTIN DOMAIN FAMILY 3, MEMBER B	-1,20	-1,61
CLEC4A2	C-TYPE LECTIN DOMAIN FAMILY 4, MEMBER A2	1,75	1,41

CLEC4A3	C-TYPE LECTIN DOMAIN FAMILY 4, MEMBER A3	1,81	1,46
CLEC4B1	C-TYPE LECTIN DOMAIN FAMILY 4, MEMBER B	1,90	1,53
CLEC4D	C-TYPE LECTIN DOMAIN FAMILY 4, MEMBER D	2,11	1,93
CLEC4E	C-TYPE LECTIN DOMAIN FAMILY 4, MEMBER E	1,46	2,62
CLEC4N	C-TYPE LECTIN DOMAIN FAMILY 4, MEMBER N	2,93	1,86
CLEC5A	C-TYPE LECTIN DOMAIN FAMILY 5, MEMBER A	1,60	1,42
CLEC7A	C-TYPE LECTIN DOMAIN FAMILY 7, MEMBER A	3,72	1,35
CNFN	CORNIFELIN	-1,42	-1,59
CNN1	CALPONIN 1	-1,49	-2,14
COTL1	RIKEN CDNA 2010004C08 GENE	1,89	1,43
COX7A1	CYTOCHROME C OXIDASE, SUBUNIT VIIA 1	-1,26	-1,75
COX8B	CYTOCHROME C OXIDASE, SUBUNIT VIIIB	-1,35	-1,73
CPXM1	CARBOXYPEPTIDASE X 1 (M14 FAMILY)	1,62	1,32
CRIM1	CYSTEINE RICH TRANSMEMBRANE BMP REGULATOR 1 (CHORDIN LIKE)	-1,54	-1,28
CRISPLD2	CYSTEINE-RICH SECRETORY PROTEIN LCCL DOMAIN CONTAINING 2	-1,59	-1,22
CSF2RB2	COLONY STIMULATING FACTOR 2 RECEPTOR, BETA 2, LOW-AFFINITY (GRANULOCYTE-MACROPHAGE)	2,22	1,86
CSRP3	CYSTEINE AND GLYCINE-RICH PROTEIN 3	-1,35	-1,50
CST8	CYSTATIN 8 (CYSTATIN-RELATED EPIDIDYMAL SPERMATOGENIC)	-1,82	-1,69
CSTAD	CSA-CONDITIONAL, T CELL ACTIVATION-DEPENDENT PROTEIN	1,22	-1,43
CSTB	CYSTATIN B	1,97	1,41
CTGF	CONNECTIVE TISSUE GROWTH FACTOR	-1,45	1,27
CTSC	CATHEPSIN C	2,55	1,42
CTSD	CATHEPSIN D	1,89	1,88
CTSK	CATHEPSIN K	5,00	2,09
CTSS	CATHEPSIN S	3,21	2,16
CTSZ	CATHEPSIN Z	2,21	1,57
CXCL10	CHEMOKINE (C-X-C MOTIF) LIGAND 10	6,00	2,60
CXCL13	CHEMOKINE (C-X-C MOTIF) LIGAND 13	1,94	1,72
CXCL14	CHEMOKINE (C-X-C MOTIF) LIGAND 14	-1,29	-1,66
CXCL16	CHEMOKINE (C-X-C MOTIF) LIGAND 16	1,75	1,34
CXCL5	CHEMOKINE (C-X-C MOTIF) LIGAND 5	1,72	1,40
CXCL9	CHEMOKINE (C-X-C MOTIF) LIGAND 9	4,02	3,07
CYBA	CYTOCHROME B-245, ALPHA POLYPEPTIDE	2,02	1,44
CYBB	CYTOCHROME B-245, BETA POLYPEPTIDE	2,29	1,96
CYP1A1	CYTOCHROME P450, FAMILY 1, SUBFAMILY A, POLYPEPTIDE 1	-1,74	-1,73
CYP26B1	CYTOCHROME P450, FAMILY 26, SUBFAMILY B, POLYPEPTIDE 1	1,52	1,08
CYP2A4	CYTOCHROME P450, FAMILY 2, SUBFAMILY A, POLYPEPTIDE 4	-1,74	-1,70
CYP2A5	CYTOCHROME P450, FAMILY 2, SUBFAMILY A, POLYPEPTIDE 4	-2,00	-1,79
CYP2F2	CYTOCHROME P450, FAMILY 2, SUBFAMILY F, POLYPEPTIDE 2	-2,16	-1,66
CYP4B1	CYTOCHROME P450, FAMILY 4, SUBFAMILY B, POLYPEPTIDE 1	-1,64	-1,35
CYP4F18	CYTOCHROME P450, FAMILY 4, SUBFAMILY F, POLYPEPTIDE 18	1,94	1,48
CYS1	CYSTIN 1	-1,53	1,17
D	46	A	B
D030029J20RIK	RIKEN CDNA D030029J20 GENE	-1,61	-1,22
D11ERTD759E	RNF213 RING FINGER PROTEIN 213	2,05	1,21
D11LGP2E	DEXH, (ASP-GLU-X-HIS) BOX POLYPEPTIDE 58	1,74	1,39
D14ERTD668E	DNA SEGMENT, CHR 14, ERATO DOI 668, EXPRESSED	2,99	1,59
D17H6S56E-5	DNA SEGMENT, CHR 17, HUMAN D6S56E 5	2,04	1,89
D2ERTD750E	DNA SEGMENT, CHR 2, ERATO DOI 750, EXPRESSED	1,44	1,66
DAXX	FAS DEATH DOMAIN-ASSOCIATED PROTEIN	1,50	1,19
DBP	D SITE ALBUMIN PROMOTER BINDING PROTEIN	-1,35	1,30
DEPDC1B	DEP DOMAIN CONTAINING 1B	1,62	1,44
DMN	DESMUSLIN	-1,48	-1,55
EAR1	EOSINOPHIL-ASSOCIATED, RIBONUCLEASE A FAMILY, MEMBER 1	1,51	1,19
EAR6	EOSINOPHIL-ASSOCIATED, RIBONUCLEASE A FAMILY, MEMBER 6	1,73	1,91
EBI3	EPSTEIN-BARR VIRUS INDUCED GENE 3	1,52	1,94
ECT2	ECT2 ONCOGENE	1,55	1,28
EDG1	ENDOTHELIAL DIFFERENTIATION SPHINGOLIPID G-PROTEIN-COUPLED RECEPTOR 1	-1,59	-1,31
EFNB1	EPHRIN B1	-1,57	-1,32
EG630499	SIMILAR TO HISTOCOMPATIBILITY 2, Q REGION LOCUS 10	1,89	1,70
EIF2AK2	EUKARYOTIC TRANSLATION INITIATION FACTOR 2-ALPHA KINASE 2	1,78	1,42
EMILIN2	ELASTIN MICROFIBRIL INTERFACER 2	1,34	1,54
EMR1	EGF-LIKE MODULE CONTAINING, MUCIN-LIKE, HORMONE RECEPTOR-LIKE SEQUENCE 1	1,79	1,60
EPAS1	ENDOTHELIAL PAS DOMAIN PROTEIN 1	-1,63	-1,49
EPSTI1	RIKEN CDNA 5033415K03 GENE	1,93	1,38
ERDR1	ERYTHROID DIFFERENTIATION REGULATOR 1	-1,53	-1,34
ERRFI1	ERBB RECEPTOR FEEDBACK INHIBITOR 1	-1,70	-1,28
ES22	ESTERASE 22	-1,58	-1,49
ESM1	ENDOTHELIAL CELL-SPECIFIC MOLECULE 1	-1,69	-1,70
EVI2A	ECOTROPIC VIRAL INTEGRATION SITE 2A	2,02	1,45
EXO1	EXONUCLEASE 1	1,55	1,58
F10	COAGULATION FACTOR X	3,00	2,99
F7	COAGULATION FACTOR VII	1,74	2,08
FABP1	FATTY ACID BINDING PROTEIN 1, LIVER	-1,85	-1,77
FABP3	FATTY ACID BINDING PROTEIN 3, MUSCLE AND HEART	-1,76	-1,62
FBXO39	F-BOX PROTEIN 39	2,43	1,37
FBXO5	F-BOX ONLY PROTEIN 5	1,66	1,52
FCER1G	FC RECEPTOR, IGE, HIGH AFFINITY I, GAMMA POLYPEPTIDE	2,25	1,71
FCGR1	FC RECEPTOR, IGG, HIGH AFFINITY I	3,67	2,51
FCGR2B	FC RECEPTOR, IGG, LOW AFFINITY IIB	2,22	1,93
FCGR3	FC RECEPTOR, IGG, LOW AFFINITY III	2,39	2,21
FCGR3A	FC FRAGMENT OF IGG, LOW AFFINITY IIIA, RECEPTOR	2,00	1,98
FEN1	FLAP STRUCTURE SPECIFIC ENDONUCLEASE 1	1,74	1,47
FFAR2	FREE FATTY ACID RECEPTOR 2	2,12	1,39
FHL1	FOUR AND A HALF LIM DOMAINS 1	-1,60	-1,46

FHL2	FOUR AND A HALF LIM DOMAINS 2	-1,50	-1,65
FMO3	FLAVIN CONTAINING MONOOXYGENASE 3	-1,89	-1,33
FOS	FBJ OSTEOSARCOMA ONCOGENE	-1,27	1,26
FOXM1	FORKHEAD BOX M1	1,80	1,37
FPR1	FORMYL PEPTIDE RECEPTOR 1	1,48	2,33
FPR-RS2	FORMYL PEPTIDE RECEPTOR, RELATED SEQUENCE 2	2,15	3,15
FUT7	FUCOSYLTRANSFERASE 7	1,51	1,31
FXYD4	RIKEN CDNA 0610008I02 GENE	1,22	1,87
GALNTL2	UDP-N-ACETYL-ALPHA-D-GALACTOSAMINE:POLYPEPTIDE N-ACETYLGALACTOSAMINYLTRANSFERASE-LIKE 2	-1,57	-1,03
GAS2L3	GROWTH ARREST-SPECIFIC 2 LIKE 3	1,34	1,62
GAS6	GROWTH ARREST SPECIFIC 6	-1,51	-1,27
GATA2	GATA BINDING PROTEIN 2	-1,60	-1,36
GATM	GLYCINE AMIDINOTRANSFERASE (L-ARGININE:GLYCINE AMIDINOTRANSFERASE)	2,10	1,53
GBP2	GUANYLATE NUCLEOTIDE BINDING PROTEIN 2	1,49	1,58
GBP3	GUANYLATE NUCLEOTIDE BINDING PROTEIN 4	2,23	1,53
GBP4	MACROPHAGE ACTIVATION 2	1,31	1,58
GBP5	GUANYLATE NUCLEOTIDE BINDING PROTEIN 5	1,55	1,56
GINS1	RIKEN CDNA 2810418N01 GENE	1,74	1,45
GLRX	GLUTAREDOXIN	1,69	1,24
GM1960	GENE MODEL 1960	1,90	1,32
GMNN	GEMININ	1,56	1,27
GPNMB	GLYCOPROTEIN (TRANSMEMBRANE) NMB	1,77	1,86
GPR109A	G PROTEIN-COUPLED RECEPTOR 109A	1,81	1,11
GPR116	G PROTEIN-COUPLED RECEPTOR 116	-1,59	-1,57
GPR162	G PROTEIN-COUPLED RECEPTOR 162	1,59	1,30
GPR171	G PROTEIN-COUPLED RECEPTOR 171	1,24	1,57
GPR176	G PROTEIN-COUPLED RECEPTOR 176	1,55	1,37
GPR65	G-PROTEIN COUPLED RECEPTOR 65	1,56	1,86
GRIA1	GLUTAMATE RECEPTOR, IONOTROPIC, AMPA1 (ALPHA 1)	-1,62	-1,32
GSN	GELSOLIN	-1,53	-1,07
GSTA2	GLUTATHIONE S-TRANSFERASE, ALPHA 2 (YC2)	-1,75	-1,52
GSTA3	GLUTATHIONE S-TRANSFERASE, ALPHA 3	-2,03	-1,32
GSTM1	GLUTATHIONE S-TRANSFERASE, MU 1	-1,79	-1,48
GSTM3	GLUTATHIONE S-TRANSFERASE, MU 3	-1,81	-1,27
GVIN1	GTPASE, VERY LARGE INTERFERON INDUCIBLE 1	1,51	1,26
H2-AA	HISTOCOMPATIBILITY 2, CLASS II ANTIGEN A, ALPHA	1,70	1,64
H2-AB1	HISTOCOMPATIBILITY 2, CLASS II ANTIGEN A, BETA 1	1,94	1,58
H2AFX	H2A HISTONE FAMILY, MEMBER X	1,63	1,51
H2-D4	HISTOCOMPATIBILITY 2, D REGION LOCUS 4	1,82	1,74
H2-DMA	HISTOCOMPATIBILITY 2, CLASS II, LOCUS DMA	1,54	1,52
H2-EB1	HISTOCOMPATIBILITY 2, CLASS II ANTIGEN E BETA	1,65	1,51
H2-K1	HISTOCOMPATIBILITY 2, K1, K REGION	1,82	1,52
H2-Q2	HISTOCOMPATIBILITY 2, Q REGION LOCUS 2	1,96	1,53
H2-Q5	HISTOCOMPATIBILITY 2, Q REGION LOCUS 5	1,88	1,43
H2-Q7	HISTOCOMPATIBILITY 2, Q REGION LOCUS 7	2,00	1,55
H2-Q8	HISTOCOMPATIBILITY 2, Q REGION LOCUS 8	2,48	1,63
H2-T22	HISTOCOMPATIBILITY 2, T REGION LOCUS 22	2,08	1,65
H2-T23	HISTOCOMPATIBILITY 2, T REGION LOCUS 23	2,08	1,52
HCK	HEMOPOIETIC CELL KINASE	1,79	1,52
HCLS1	HEMATOPOIETIC CELL SPECIFIC LYN SUBSTRATE 1	1,63	1,31
HEBP1	HEME BINDING PROTEIN 1	1,53	1,27
HERPUD1	HOMOCYSTEINE-INDUCIBLE, ENDOPLASMIC RETICULUM STRESS-INDUCIBLE, UBIQUITIN-LIKE DOMAIN MEMBER 1	-1,60	-1,14
HEXA	HEXOSAMINIDASE A	1,53	1,50
HEXB	HEXOSAMINIDASE B	1,87	1,37
HEY1	HAIRY/ENHANCER-OF-SPLIT RELATED WITH YRPW MOTIF 1	-1,64	-1,48
HIST1H1B	HISTONE 1, H1B	2,66	1,86
HIST1H2AA	HISTONE 1, H2AA	1,98	1,50
HIST1H2AB	HISTONE CLUSTER 1, H2AB	2,94	2,08
HIST1H2AF	HISTONE 1, H2AF	1,99	1,63
HIST1H2AI	HISTONE CLUSTER 1, H2AI	2,41	1,73
HIST1H2AK	HISTONE 1, H2AK	2,69	2,32
HIST2H2AC	HISTONE 2, H2AB	1,77	1,51
HK3	HEXOKINASE 3	1,72	1,80
HMGB2	HIGH MOBILITY GROUP BOX 2	1,77	1,78
HMGN2	HIGH-MOBILITY GROUP NUCLEOSOMAL BINDING DOMAIN 2	1,51	1,18
HMMR	HYALURONAN MEDIATED MOTILITY RECEPTOR (RHAMM)	1,58	1,46
HMOX1	HEME OXYGENASE (DECYCLING) 1	1,57	1,50
HRC	HISTIDINE RICH CALCIUM BINDING PROTEIN	-1,46	-1,85
HSP110	HEAT SHOCK PROTEIN 110	-1,55	-1,42
HSPA1A	HEAT SHOCK PROTEIN 1A	-1,95	-1,50
IFI204	INTERFERON ACTIVATED GENE 204	2,82	1,70
IFI27	INTERFERON, ALPHA-INDUCIBLE PROTEIN 27	5,66	2,67
IFI30	INTERFERON GAMMA INDUCIBLE PROTEIN 30	2,16	1,64
IFI44	INTERFERON-INDUCED PROTEIN 44	2,29	1,28
IFIH1	INTERFERON INDUCED WITH HELICASE C DOMAIN 1	1,64	1,24
IFIT1	INTERFERON-INDUCED PROTEIN WITH TETRATRICOPEPTIDE REPEATS 1	4,11	1,58
IFIT2	INTERFERON-INDUCED PROTEIN WITH TETRATRICOPEPTIDE REPEATS 2	2,56	1,42
IFIT3	INTERFERON-INDUCED PROTEIN WITH TETRATRICOPEPTIDE REPEATS 3	2,88	1,62
IFITM6	INTERFERON INDUCED TRANSMEMBRANE PROTEIN 6	1,14	1,67
IGSF6	IMMUNOGLOBULIN SUPERFAMILY, MEMBER 6	1,41	1,92
IGTP	INTERFERON GAMMA INDUCED GTPASE	1,79	1,57
IIGP1	INTERFERON INDUCIBLE GTPASE 1	1,91	1,63
IL10RA	INTERLEUKIN 10 RECEPTOR, ALPHA	1,67	1,41
IL18BP	INTERLEUKIN 18 BINDING PROTEIN	2,27	1,73
IL1RN	INTERLEUKIN 1 RECEPTOR ANTAGONIST	1,89	1,19

IL4I1	INTERLEUKIN 4 INDUCED 1	1,66	1,20
IMPA2	INOSITOL (MYO)-1(OR 4)-MONOPHOSPHATASE 2	1,59	1,68
INCENP	INNER CENTROMERE PROTEIN	2,04	1,67
INHBA	INHIBIN BETA-A	1,88	1,62
INMT	INDOLETHYLAMINE N-METHYLTRANSFERASE	-1,66	-1,18
IRF5	INTERFERON REGULATORY FACTOR 5	1,82	1,41
IRF7	INTERFERON REGULATORY FACTOR 7	6,15	2,49
IRG1	IMMUNORESPONSIVE GENE 1	5,76	2,64
IRGM	IMMUNITY-RELATED GTPASE FAMILY, M	1,57	1,11
ISG15	INTERFERON, ALPHA-INDUCIBLE PROTEIN	6,50	2,28
ISG20	INTERFERON-STIMULATED PROTEIN	1,54	1,18
ITGB2	INTEGRIN BETA 2	1,72	1,35
KCNA1	POTASSIUM VOLTAGE-GATED CHANNEL, SHAKER-RELATED SUBFAMILY, MEMBER 1	-1,19	-1,51
KIF20A	KINESIN FAMILY MEMBER 20A	4,32	2,20
KIF22	EXPRESSED SEQUENCE AL033313	3,54	1,96
KIF23	KINESIN FAMILY MEMBER 23	1,52	1,38
KIF4	KINESIN FAMILY MEMBER 4	1,75	1,39
KIFC1	KINESIN FAMILY MEMBER C1	2,10	1,86
KLF15	KRUPPEL-LIKE FACTOR 15	-1,64	-1,28
KLF7	KRUPPEL-LIKE FACTOR 7 (UBIQUITOUS)	-1,57	-1,18
KPNA2	KARYOPHERIN (IMPORTIN) ALPHA 2	1,50	1,32
KRT15	KERATIN COMPLEX 1, ACIDIC, GENE 15	-1,29	-2,71
KRT5	RIKEN CDNA 3300001P10 GENE	1,12	-1,47
KRT79	CDNA SEQUENCE BC031593	1,73	1,27
LAIR1	LEUKOCYTE-ASSOCIATED IG-LIKE RECEPTOR 1	1,85	1,56
LAPTM5	LYSOSOMAL-ASSOCIATED PROTEIN TRANSMEMBRANE 5	1,50	1,49
LCN2	LIPOCALIN 2	5,51	4,27
LCP2	LYMPHOCYTE CYTOSOLIC PROTEIN 2	1,36	1,56
LGALS1	LECTIN, GALACTOSE BINDING, SOLUBLE 1	1,60	1,34
LGALS3	LECTIN, GALACTOSE BINDING, SOLUBLE 3	1,96	1,91
LGALS3BP	LECTIN, GALACTOSIDE-BINDING, SOLUBLE, 3 BINDING PROTEIN	3,61	1,77
LGMN	LEGUMAIN	1,87	1,65
LILRB3	LEUKOCYTE IMMUNOGLOBULIN-LIKE RECEPTOR, SUBFAMILY B (WITH TM AND ITIM DOMAINS), MEMBER 3	1,51	2,13
LILRB4	LEUKOCYTE IMMUNOGLOBULIN-LIKE RECEPTOR, SUBFAMILY B, MEMBER 4	2,71	1,87
LIPF	LIPASE, GASTRIC	1,55	1,24
LMNB1	LAMIN B1	1,55	1,61
LOC547343	H2-D1, HISTOCOMPATIBILITY 2, D REGION LOCUS 1	1,97	1,69
LOC56628	H2-K1, HISTOCOMPATIBILITY 2, K1, K REGION	1,72	1,51
LOC619863	GM6105, PREDICTED GENE 6105	1,74	1,72
LOC640374	SIMILAR TO GLYCERALDEHYDE-3-PHOSPHATE DEHYDROGENASE (GAPDH)	1,54	1,25
LOC667373	GM14446, PREDICTED GENE 14446	1,53	1,13
LOC668856	STARD9, START DOMAIN CONTAINING 9	-1,59	-1,36
LOC677118	(NO GENE)	-1,39	-1,63
LPL	LIPOPROTEIN LIPASE	1,58	1,24
LPXN	LEUPAXIN	1,84	1,47
LRG1	LEUCINE-RICH ALPHA-2-GLYCOPROTEIN 1	1,80	1,65
LRRC25	LEUCINE RICH REPEAT CONTAINING 25	1,44	1,51
LST1	LEUKOCYTE SPECIFIC TRANSCRIPT 1	1,69	1,47
LTBP4	RIKEN CDNA 2310046A13 GENE	-1,61	-1,31
LY6A	LYMPHOCYTE ANTIGEN 6 COMPLEX, LOCUS A	1,50	1,80
LY6C	LYMPHOCYTE ANTIGEN 6 COMPLEX, LOCUS C	1,38	1,96
LY6F	LYMPHOCYTE ANTIGEN 6 COMPLEX, LOCUS F	8,89	4,89
LY6I	LYMPHOCYTE ANTIGEN 6 COMPLEX, LOCUS I	2,35	3,46
LY86	LYMPHOCYTE ANTIGEN 86	2,13	1,90
LY9	LYMPHOCYTE ANTIGEN 9	1,43	1,58
LYPD2	LY6/PLAUR DOMAIN CONTAINING 2	-1,47	-1,62
LZP-S	LYZ1, LYSOZYME 1	1,56	1,42
MAD2L1	MAD2 (MITOTIC ARREST DEFICIENT, HOMOLOG)-LIKE 1 (YEAST)	1,84	1,49
MALAT1	METASTASIS ASSOCIATED LUNG ADENOCARCINOMA TRANSCRIPT 1 (NON-CODING RNA)	-1,63	-1,69
MAPT	MICROTUBULE-ASSOCIATED PROTEIN TAU	-1,69	-1,37
MARCO	MACROPHAGE RECEPTOR WITH COLLAGENOUS STRUCTURE	2,82	1,86
MAT2A	METHIONINE ADENOSYLTRANSFERASE II, ALPHA	-1,53	-1,18
MB	MYOGLOBIN	-1,67	-1,46
MCM10	MINICHROMOSOME MAINTENANCE DEFICIENT 10 (S. CEREVISIAE)	1,76	1,75
MCM2	MINICHROMOSOME MAINTENANCE DEFICIENT 2 MITOTIN (S. CEREVISIAE)	1,29	1,67
MCM3	MINICHROMOSOME MAINTENANCE DEFICIENT 3 (S. CEREVISIAE)	1,51	1,34
MCM5	MINICHROMOSOME MAINTENANCE DEFICIENT 5, CELL DIVISION CYCLE 46 (S. CEREVISIAE)	1,62	1,47
MCM6	MINICHROMOSOME MAINTENANCE DEFICIENT 6 (MIS5 HOMOLOG, S. POMBE) (S. CEREVISIAE)	1,51	1,33
MEFV	MEDITERRANEAN FEVER	1,52	1,45
MFSD2	MAJOR FACILITATOR SUPERFAMILY DOMAIN CONTAINING 2	-1,59	-1,42
MGC73635	SIMILAR TO HISTONE 2A	3,89	2,38
MKI67	ANTIGEN IDENTIFIED BY MONOCLONAL ANTIBODY KI 67	2,22	2,51
MLKL	MIXED LINEAGE KINASE DOMAIN-LIKE	1,71	1,12
MMP12	MATRIX METALLOPEPTIDASE 12	1,31	1,58
MMP14	MATRIX METALLOPEPTIDASE 14 (MEMBRANE-INSERTED)	1,59	1,49
MMP8	MATRIX METALLOPEPTIDASE 8	1,26	3,41
MNDA	MYELOID CELL NUCLEAR DIFFERENTIATION ANTIGEN	2,33	1,58
MPA2L	MACROPHAGE ACTIVATION 2 LIKE	1,55	1,41
MPEG1	MACROPHAGE EXPRESSED GENE 1	1,77	1,59
MPO	MYELOPEROXIDASE	1,00	1,63
MS4A4C	MEMBRANE-SPANNING 4-DOMAINS, SUBFAMILY A, MEMBER 4C	1,66	1,32
MS4A6B	MEMBRANE-SPANNING 4-DOMAINS, SUBFAMILY A, MEMBER 6B	2,22	2,02
MS4A6C	MEMBRANE-SPANNING 4-DOMAINS, SUBFAMILY A, MEMBER 6C	2,73	2,71
MS4A6D	MEMBRANE-SPANNING 4-DOMAINS, SUBFAMILY A, MEMBER 6D	4,03	3,41
MS4A7	MEMBRANE-SPANNING 4-DOMAINS, SUBFAMILY A, MEMBER 7	5,49	3,01

MSR1	MACROPHAGE SCAVENGER RECEPTOR 1	2,17	1,85
MT2	METALLOTHIONEIN 2	1,67	1,40
MTHFD2	METHYLENETETRAHYDROFOLATE DEHYDROGENASE (NAD+ DEPENDENT), METHENYLTETRAHYDROFOLATE CYCLOHYDROLASE	-1,04	1,51
MUC5B	MUCIN 5, SUBTYPE B, TRACHEOBRONCHIAL	-1,53	-1,29
MX1	MYXOVIRUS (INFLUENZA VIRUS) RESISTANCE 1	1,51	1,08
MX2	MYXOVIRUS (INFLUENZA VIRUS) RESISTANCE 2	2,50	1,45
MXD3	MAX DIMERIZATION PROTEIN 3	1,68	1,45
MYBPHL	RIKEN CDNA 1110037P11 GENE	-1,26	-1,52
MYH6	MYOSIN, HEAVY POLYPEPTIDE 6, CARDIAC MUSCLE, ALPHA	-1,45	-1,70
MYL4	MYOSIN, LIGHT POLYPEPTIDE 4	-1,73	-1,95
MYL7	MYOSIN, LIGHT POLYPEPTIDE 7, REGULATORY	-2,06	-2,44
MYL9	MYOSIN, LIGHT POLYPEPTIDE 9, REGULATORY	-1,52	-1,44
MYO1F	MYOSIN IF	1,99	1,57
NCAPD2	RIKEN CDNA 2810406C15 GENE	1,46	1,60
NCAPG	RIKEN CDNA 5730507H05 GENE	1,93	1,71
NCAPG2	LEUCINE ZIPPER PROTEIN 5	2,37	1,62
NCF1	NEUTROPHIL CYTOSOLIC FACTOR 1	1,94	1,62
NCF2	NEUTROPHIL CYTOSOLIC FACTOR 2	1,56	1,59
NCF4	NEUTROPHIL CYTOSOLIC FACTOR 4	1,56	1,51
NGP	NEUTROPHILIC GRANULE PROTEIN	-1,02	2,20
NMI	N-MCY (AND STAT) INTERACTOR	1,53	1,37
NMRAL1	RIKEN CDNA 1110025F24 GENE	1,65	1,41
NOTCH3	NOTCH GENE HOMOLOG 3 (DROSOPHILA)	-1,69	-1,42
NOXO1	NADPH OXIDASE ORGANIZER 1	2,78	1,54
NPPA	NATRIURETIC PEPTIDE PRECURSOR TYPE A	-1,83	-2,05
NPR3	NATRIURETIC PEPTIDE RECEPTOR 3	-1,60	-1,91
NR1D2	NUCLEAR RECEPTOR SUBFAMILY 1, GROUP D, MEMBER 2	-1,62	-1,17
NUSAP1	EXPRESSED SEQUENCE AW547774	1,51	1,35
OAS1A	2'-5' OLIGOADENYLATE SYNTHETASE 1A	4,27	2,76
OAS1F	2'-5' OLIGOADENYLATE SYNTHETASE 1F	6,33	2,86
OAS2	2'-5' OLIGOADENYLATE SYNTHETASE 2	1,61	1,35
OASL1	2'-5' OLIGOADENYLATE SYNTHETASE-LIKE 1	3,35	1,60
OASL2	2'-5' OLIGOADENYLATE SYNTHETASE-LIKE 2	3,85	1,75
OGFR	OPIOID GROWTH FACTOR RECEPTOR	1,67	1,25
OGN	OSTEOGLYCIN	-1,54	-1,09
OIP5	OPA INTERACTING PROTEIN 5	1,88	1,20
ORM1	OROSOMUCOID 1	1,81	1,50
ORM2	OROSOMUCOID 2	1,72	2,20
P2RY6	PYRIMIDINERGIC RECEPTOR P2Y, G-PROTEIN COUPLED, 6	1,74	1,69
PARP9	POLY (ADP-RIBOSE) POLYMERASE FAMILY, MEMBER 9	1,74	1,59
PBK	PDZ BINDING KINASE	3,03	1,53
PCOLCE2	PROCOLLAGEN C-ENDOPEPTIDASE ENHANCER 2	-1,60	-1,19
PDGFRB	PLATELET DERIVED GROWTH FACTOR RECEPTOR, BETA POLYPEPTIDE	-1,52	-1,55
PER1	PERIOD HOMOLOG 1 (DROSOPHILA)	-1,37	1,29
PGAM2	PHOSPHOGLYCERATE MUTASE 2	-1,31	-1,60
PHF11	PHD FINGER PROTEIN 11	3,12	1,71
PIRA3	PAIRED-IG-LIKE RECEPTOR A3	1,41	1,78
PLA2G7	PHOSPHOLIPASE A2, GROUP VII (PLATELET-ACTIVATING FACTOR ACETYLHYDROLASE, PLASMA)	1,33	1,59
PLAC8	PLACENTA-SPECIFIC 8	1,43	1,62
PLAC9	PLACENTA SPECIFIC 9	-1,66	-1,21
PLD3	PHOSPHOLIPASE D FAMILY, MEMBER 3	1,50	1,33
PLD4	PHOSPHOLIPASE D FAMILY, MEMBER 4	1,63	1,30
PLEK	PLECKSTRIN	1,34	1,53
PLEKHK1	PLECKSTRIN HOMOLOGY DOMAIN CONTAINING, FAMILY K MEMBER 1	-1,54	-1,30
PLK1	POLO-LIKE KINASE 1 (DROSOPHILA)	2,14	1,91
PLUNC	PALATE, LUNG, AND NASAL EPITHELIUM CARCINOMA ASSOCIATED	-2,06	-2,66
PLVAP	PLASMALEMMA VESICLE ASSOCIATED PROTEIN	-1,52	-1,29
PMAIP1	PHORBOL-12-MYRISTATE-13-ACETATE-INDUCED PROTEIN 1	1,11	1,61
PNPLA7	PATATIN-LIKE PHOSPHOLIPASE DOMAIN CONTAINING 7	1,51	1,39
PON1	PARAOXONASE 1	-2,65	-1,43
POR	P450 (CYTOCHROME) OXIDOREDUCTASE	-1,61	-1,31
PPAP2B	PHOSPHATIDIC ACID PHOSPHATASE TYPE 2B	-1,96	-1,87
PPARG	PEROXISOME PROLIFERATOR ACTIVATED RECEPTOR GAMMA	1,53	1,10
PPFIA4	PROTEIN TYROSINE PHOSPHATASE, RECEPTOR TYPE, F POLYPEPTIDE (PTPRF), INTERACTING PROTEIN (LIPRIN), ALPHA 4	1,85	1,30
PRC1	PROTEIN REGULATOR OF CYTOKINESIS 1	3,17	1,76
PRG4	PROTEOGLYCAN 4, MEGAKARYOCYTE STIMULATING FACTOR PRECURSOR AND CARTILAGE SUPERFICIAL ZONE PROTEIN	1,55	1,01
PRICKLE1	PRICKLE LIKE 1 (DROSOPHILA)	-1,42	-1,63
PROCR	PROTEIN C RECEPTOR, ENDOTHELIAL	1,30	1,51
PROK2	PROKINETICIN 2	1,07	1,66
PSAP	PROSAPOSIN	1,91	2,08
PSMB8	PROTEOSOME (PROSOME, MACROPAIN) SUBUNIT, BETA TYPE 8 (LARGE MULTIFUNCTIONAL PEPTIDASE 7)	1,93	1,37
PSMB9	PROTEOSOME (PROSOME, MACROPAIN) SUBUNIT, BETA TYPE 9 (LARGE MULTIFUNCTIONAL PEPTIDASE 2)	1,66	1,59
PSRC1	PROLINE/SERINE-RICH COILED-COIL 1	1,65	1,20
PTPRB	PROTEIN TYROSINE PHOSPHATASE, RECEPTOR TYPE, B	-1,54	-1,49
PYCARD	PYD AND CARD DOMAIN CONTAINING	2,08	1,52
RAB32	RAB32, MEMBER RAS ONCOGENE FAMILY	2,27	1,52
RAB6B	RAB6B, MEMBER RAS ONCOGENE FAMILY	-1,55	-1,64
RAD51	RAD51 HOMOLOG (S. CEREVISIAE)	1,51	1,60
RAD51AP1	RAD51 ASSOCIATED PROTEIN 1	1,61	1,39
RAMP2	RECEPTOR (CALCITONIN) ACTIVITY MODIFYING PROTEIN 2	-1,64	-1,43
RASA4	RAS P21 PROTEIN ACTIVATOR 4	1,68	1,67
RBMS3	RNA BINDING MOTIF, SINGLE STRANDED INTERACTING PROTEIN	-1,52	-1,23
RBP4	RETINOL BINDING PROTEIN 4, PLASMA	-1,60	-1,30
RBPSUHL	RBPJL, RECOMBINATION SIGNAL BINDING PROTEIN FOR IMMUNOGLOBULIN KAPPA J REGION-LIKE	1,14	-1,34
RETNLA	RESISTIN LIKE ALPHA	3,21	3,27

RETNLG	RESISTIN LIKE GAMMA	-1,60	1,48
RFC5	REPLICATION FACTOR C (ACTIVATOR 1) 5	1,74	1,41
RGS1	REGULATOR OF G-PROTEIN SIGNALING 1	2,24	2,01
RGS10	REGULATOR OF G-PROTEIN SIGNALLING 10	1,55	1,31
RGS16	REGULATOR OF G-PROTEIN SIGNALING 16	1,29	1,68
RNF149	RING FINGER PROTEIN 149	1,65	1,33
ROBO4	ROUNDABOUT HOMOLOG 4 (DROSOPHILA)	-1,58	-1,27
ROPN1L	ROPPORIN 1-LIKE	1,12	1,51
RRM2	RIBONUCLEOTIDE REDUCTASE M2	1,58	1,56
RSAD2	RADICAL S-ADENOSYL METHIONINE DOMAIN CONTAINING 2	2,52	1,92
RTP4	RECEPTOR TRANSPORTER PROTEIN 4	3,43	1,59
RXRG	RETINOID X RECEPTOR GAMMA	1,19	-1,33
S100A14	S100 CALCIUM BINDING PROTEIN A14	1,90	1,26
S100A4	S100 CALCIUM BINDING PROTEIN A4	1,34	1,51
S100A8	S100 CALCIUM BINDING PROTEIN A8 (CALGRANULIN A)	-1,28	3,52
S100A9	S100 CALCIUM BINDING PROTEIN A9 (CALGRANULIN B)	-1,41	2,88
SAA1	SERUM AMYLOID A 1	3,82	1,89
SAA2	SERUM AMYLOID A 2	1,51	1,50
SAA3	SERUM AMYLOID A 3	14,96	7,74
SAMSN1	SAM DOMAIN, SH3 DOMAIN AND NUCLEAR LOCALIZATION SIGNALS, 1	1,67	1,47
SCD2	STEAROYL-COENZYME A DESATURASE 2	-1,59	-1,19
SCGB3A1	SECRETOGLOBIN, FAMILY 3A, MEMBER 1	-1,33	-1,54
SDPR	SERUM DEPRIVATION RESPONSE	-1,55	-1,25
SELENBP1	SELENIUM BINDING PROTEIN 1	-1,74	-1,33
SELENBP2	SELENIUM BINDING PROTEIN 2	-1,66	-1,24
SELPL	SELECTIN, PLATELET (P-SELECTIN) LIGAND	1,63	1,38
SEMA3G	SEMA DOMAIN, IMMUNOGLOBULIN DOMAIN (IG), SHORT BASIC DOMAIN, SECRETED, (SEMAPHORIN) 3G	-1,59	-1,57
SERPINA3F	CDNA SEQUENCE BC049975	1,17	1,69
SERPINA3G	SERINE (OR CYSTEINE) PEPTIDASE INHIBITOR, CLADE A, MEMBER 3G	1,66	2,67
SGOL1	SHUGOSHIN-LIKE 1 (S. POMBE)	2,26	1,94
SGOL2	SHUGOSHIN-LIKE 2 (S. POMBE)	1,88	1,45
SHCBP1	SHC SH2-DOMAIN BINDING PROTEIN 1	1,46	1,54
SIGLEC1	SIALIC ACID BINDING IG-LIKE LECTIN 1, SIALOADHESIN	1,75	1,25
SIRPA	PROTEIN TYROSINE PHOSPHATASE, NON-RECEPTOR TYPE SUBSTRATE 1	1,61	1,15
SIRPB1	SIRP-BETA B	2,12	1,27
SLA	SRC-LIKE ADAPTOR	1,61	1,52
SLAMF7	SLAM FAMILY MEMBER 7	1,73	1,44
SLAMF9	RIKEN CDNA 2310026I04 GENE	2,56	1,62
SLC11A1	SOLUTE CARRIER FAMILY 11 (PROTON-COUPLED DIVALENT METAL ION TRANSPORTERS), MEMBER 1	1,56	1,79
SLC15A3	SOLUTE CARRIER FAMILY 15, MEMBER 3	1,72	1,37
SLC16A11	SOLUTE CARRIER FAMILY 16 (MONOCARBOXYLIC ACID TRANSPORTERS), MEMBER 11	-1,81	-1,57
SLC26A4	SOLUTE CARRIER FAMILY 26, MEMBER 4	4,10	1,91
SLC2A4	SOLUTE CARRIER FAMILY 2 (FACILITATED GLUCOSE TRANSPORTER), MEMBER 4	1,29	-1,28
SLC37A2	SOLUTE CARRIER FAMILY 37 (GLYCEROL-3-PHOSPHATE TRANSPORTER), MEMBER 2	1,61	1,52
SLC7A10	SOLUTE CARRIER FAMILY 7 (CATIONIC AMINO ACID TRANSPORTER, Y+ SYSTEM), MEMBER 10	-1,49	-1,65
SLC7A2	SOLUTE CARRIER FAMILY 7 (CATIONIC AMINO ACID TRANSPORTER, Y+ SYSTEM), MEMBER 2	1,74	1,30
SLC7A8	SOLUTE CARRIER FAMILY 7 (CATIONIC AMINO ACID TRANSPORTER, Y+ SYSTEM), MEMBER 8	2,36	1,37
SLFN10	SCHLAFEN 10	2,64	1,57
SLFN2	SCHLAFEN 2	2,34	1,87
SLFN4	SCHLAFEN 4	1,75	1,85
SMAD6	MAD HOMOLOG 6 (DROSOPHILA)	-1,38	-1,65
SMPX	SMALL MUSCLE PROTEIN, X-LINKED	-1,41	-1,77
SOX18	SRY-BOX CONTAINING GENE 18	-1,33	-1,61
SOX7	SRY-BOX CONTAINING GENE 7	-1,49	-1,66
SPAG5	SPERM ASSOCIATED ANTIGEN 5	1,69	1,51
SPBC25	SPC25, NDC80 KINETOCHORE COMPLEX COMPONENT, HOMOLOG (S. CEREVISIAE)	2,53	1,61
SPINK5	SERINE PEPTIDASE INHIBITOR, KAZAL TYPE 5	1,90	1,79
SPON2	SPONDIN 2, EXTRACELLULAR MATRIX PROTEIN	1,01	-1,81
SPP1	SECRETED PHOSPHOPROTEIN 1	1,55	1,52
STAT1	SIGNAL TRANSDUCER AND ACTIVATOR OF TRANSCRIPTION 1	1,90	1,31
STAT2	SIGNAL TRANSDUCER AND ACTIVATOR OF TRANSCRIPTION 2	1,75	1,07
SULT1D1	SULFOTRANSFERASE FAMILY 1D, MEMBER 1	-1,55	-1,53
SYCE2	SYNAPTONEMAL COMPLEX CENTRAL ELEMENT PROTEIN 2	1,77	1,48
T2BP	TIFA, TRAF-INTERACTING PROTEIN WITH FORKHEAD-ASSOCIATED DOMAIN	1,23	1,69
TACC3	TRANSFORMING, ACIDIC COILED-COIL CONTAINING PROTEIN 3	2,36	1,66
TAP1	TRANSPORTER 1, ATP-BINDING CASSETTE, SUB-FAMILY B (MDR/TAP)	1,86	1,74
TBXAS1	THROMBOXANE A SYNTHASE 1, PLATELET	1,56	1,45
TCF19	TRANSCRIPTION FACTOR 19	1,85	1,59
TEK	ENDOTHELIAL-SPECIFIC RECEPTOR TYROSINE KINASE	-1,66	-1,46
TFF2	TREFOIL FACTOR 2 (SPASMOLYTIC PROTEIN 1)	1,14	1,69
TGFBI	TRANSFORMING GROWTH FACTOR, BETA INDUCED	3,32	2,41
THRSP	THYROID HORMONE RESPONSIVE SPOT14 HOMOLOG (RATTUS)	2,11	-1,17
TIMP1	TISSUE INHIBITOR OF METALLOPROTEINASE 1	1,78	1,06
TIMP3	TISSUE INHIBITOR OF METALLOPROTEINASE 3	-1,58	-1,30
TK1	THYMIDINE KINASE 1	2,34	1,68
TLR2	TOLL-LIKE RECEPTOR 2	2,40	1,66
TLR6	TOLL-LIKE RECEPTOR 6	1,20	1,51
TLR7	TOLL-LIKE RECEPTOR 7	2,02	1,45
TMEM46	TRANSMEMBRANE PROTEIN 46	1,07	-1,40
TMEM86A	TRANSMEMBRANE PROTEIN 86A	1,35	1,63
TNFAIP3	TUMOR NECROSIS FACTOR, ALPHA-INDUCED PROTEIN 3	-1,01	1,58
TNFAIP8L1	TUMOR NECROSIS FACTOR, ALPHA-INDUCED PROTEIN 8-LIKE 1	1,62	1,21
TNFAIP8L2	TUMOR NECROSIS FACTOR, ALPHA-INDUCED PROTEIN 8-LIKE 2	1,42	1,61
TNFRSF1B	TUMOR NECROSIS FACTOR RECEPTOR SUPERFAMILY, MEMBER 1B	1,85	1,39
TNFSF12-TNFSF1	TUMOR NECROSIS FACTOR (LIGAND) SUPERFAMILY, MEMBER 12-MEMBER 13	1,50	1,31

TNNI3	TROPONIN I, CARDIAC	-1,76	-1,89
TOP2A	TOPOISOMERASE (DNA) II ALPHA	1,41	1,64
TPM1	TROPOMYOSIN 1, ALPHA	-1,19	-1,56
TPX2	TPX2, MICROTUBULE-ASSOCIATED PROTEIN HOMOLOG (XENOPUS LAEVIS)	2,07	1,68
TRAIP	TRAF-INTERACTING PROTEIN	1,60	1,38
TREM2	TRIGGERING RECEPTOR EXPRESSED ON MYELOID CELLS 2C	4,14	3,99
TREM3	TRIGGERING RECEPTOR EXPRESSED ON MYELOID CELLS 3	1,05	1,78
TREX1	THREE PRIME REPAIR EXONUCLEASE 1	2,34	1,41
TRIM30	TRIPARTITE MOTIF PROTEIN 30	2,38	1,49
TRIP13	THYROID HORMONE RECEPTOR INTERACTOR 13	1,60	1,83
TRPV2	TRANSIENT RECEPTOR POTENTIAL CATION CHANNEL, SUBFAMILY V, MEMBER 2	1,58	1,37
TSPAN13	TETRASPANIN 13	-1,45	-1,59
TSPAN2	TETRASPANIN 2	-1,53	-1,20
TXNIP	THIOREDOXIN INTERACTING PROTEIN	-1,29	-1,51
TYKI	THYMIDYLATE KINASE FAMILY LPS-INDUCIBLE MEMBER	1,62	1,24
TYMS-PS	THYMIDYLATE SYNTHASE, PSEUDOGENE	1,54	1,47
TYROBP	TYRO PROTEIN TYROSINE KINASE BINDING PROTEIN	1,61	1,50
U46068	CDNA SEQUENCE U46068	-1,31	-1,54
UBE2C	UBIQUITIN-CONJUGATING ENZYME E2C	2,09	1,82
UCP1	UNCOUPLING PROTEIN 1 (MITOCHONDRIAL, PROTON CARRIER)	1,20	1,57
UHRF1	UBIQUITIN-LIKE, CONTAINING PHD AND RING FINGER DOMAINS, 1	2,17	1,72
UNG2	UNG, URACIL DNA GLYCOSYLASE	1,52	1,58
USP18	UBIQUITIN SPECIFIC PEPTIDASE 18	3,28	1,38
VAV1	VAV 1 ONCOGENE	1,95	1,47
VGLL3	VESTIGIAL LIKE 3 (DROSOPHILA)	-1,58	-1,29
VNN1	VANIN 1	2,35	1,85
VNN3	VANIN 3	1,50	1,75
XCL1	CHEMOKINE (C MOTIF) LIGAND 1	1,55	1,58
ZBP1	Z-DNA BINDING PROTEIN 1	4,19	2,03
ZMYND15	ZINC FINGER, MYND-TYPE CONTAINING 15	1,72	1,25
